# High unexpected genetic diversity of a narrow endemic terrestrial mollusc

**DOI:** 10.7717/peerj.3069

**Published:** 2017-03-16

**Authors:** Pedro M. Madeira, Rosa M. Chefaoui, Regina L. Cunha, Francisco Moreira, Susana Dias, Gonçalo Calado, Rita Castilho

**Affiliations:** 1 CCMAR, Centre for Marine Sciences, Campus de Gambelas, Faro, Portugal; 2 Departamento de Ciências da Vida, Escola de Psicologia e Ciências da Vida, Universidade Lusófona, Campo Grande, Lisboa, Portugal; 3 REN Biodiversity Chair, CIBIO/InBIO Associate Laboratory, Universidade do Porto, Campus Agrário de Vairão, Vairão, Portugal; 4 Centro de Ecologia Aplicada Prof. Baeta Neves/InBIO Associate Laboratory, Instituto Superior de Agronomia, Universidade de Lisboa, Tapada da Ajuda, Lisboa, Portugal

**Keywords:** Endemic species, Terrestrial gastropods

## Abstract

The Iberian Peninsula has an extensive record of species displaying strong genetic structure as a result of their survival in isolated pockets throughout the Pleistocene ice ages. We used mitochondrial and nuclear sequence data to analyze phylogeographic patterns in endemic land snails from a valley of central Portugal (Vale da Couda), putatively assigned to* Candidula coudensis*, that show an exceptionally narrow distributional range. The genetic survey presented here shows the existence of five main mitochondrial lineages in Vale da Couda that do not cluster together suggesting independent evolutionary histories. Our results also indicate a departure from the expectation that species with restricted distributions have low genetic variability. The putative past and contemporary models of geographic distribution of Vale da Couda lineages are compatible with a scenario of species co-existence in more southern locations during the last glacial maximum (LGM) followed by a post-LGM northern dispersal tracking the species optimal thermal, humidity and soil physical conditions.

## Introduction

Phylogeography combines evidence from both population genetics and phylogenetics, to understand the evolutionary processes that shape geographic population structure (Avise, 2000). These evolutionary processes include divergence among gene pools, demographic changes in populations, and migrations between metapopulations, generally promoted or constrained by geological and/or climate events. If genetic isolation is in place by whatever mechanism (e.g., allopatry or sexual selection), it is possible that, in time, local variants of a species turn into endemic species. Endemic species are usually found in relatively small areas (Gaston, 1994), occupying specialized habitats with small population sizes that are more susceptible to local extinctions (Primack, 2006). Endemic species therefore constitute a model to explore population genetics in what effectively can be seen as an island setting. The geographic and demographic components interact with the genetic dynamics of the species, often determining species viability. Genetic diversity is essential to ensure that populations can withstand environmental fluctuations during short timeframes and also serves as the basis for selection and capacity to adapt to changes in the environment in the long run (Frankham, 2005; Laikre et al., 2009). It is therefore important to assess the genetic properties of the populations of those species, such as genetic diversity and connectivity, as well as historical demography.

Identifying the drivers of geographic distribution patterns is also essential to understand the population dynamics in space and time. Species distribution modeling (SDM) allows one to examine the relationship between the identified presence records of a species, lineage or related species, with the environmental characteristics of these locations. From the inferred relationship it is possible to estimate the response, function and contribution of environmental variables (Austin et al., 2006), and predict the potential geographical range (Elith & Leathwick, 2009). Recently, there has been a growing trend towards the integration of SDM hindcasts with phylogeography as a useful approach to obtain consistent eco-evolutionary hypotheses. This combination allows insights into how the distribution of climatic refuges and postglacial colonization pathways may have influenced genetic diversity of current populations (see e.g., Hewitt, 2004).

Land snails are good models for evolutionary studies, since phylogeographic patterns are often preserved due to their limited dispersal capabilities and specific habitat requirements (Pfenninger, Nowak & Magnin, 2007). Also, snails display an unusually high intraspecific genetic variation, ca. 10–30% in mtDNA sequences (Bond et al., 2001; Hayashi & Chiba, 2000; Pinceel et al., 2005; Shimizu & Ueshima, 2000), which renders the taxa appropriate to understand processes shaping the partitioning of genetic variation in space. Additionally, many land snail examples in the literature show the existence of cryptic species in sympatry (Köhler & Burghardt, 2015).

**Figure 1:**
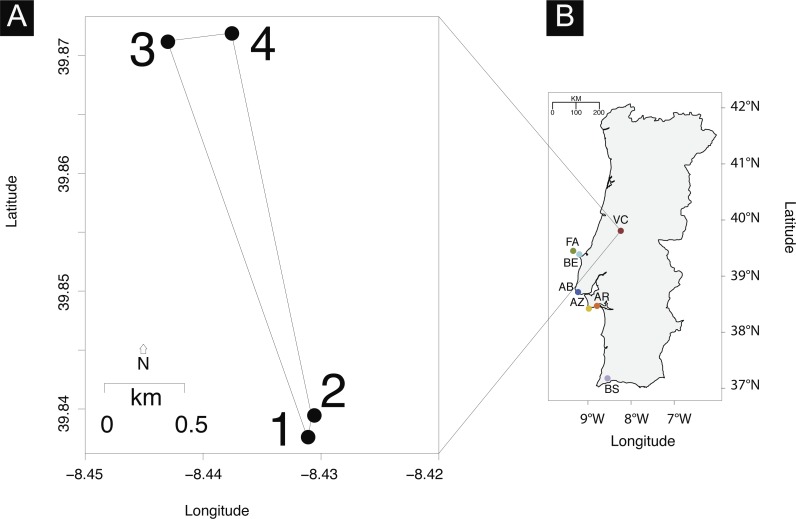
Sampling sites. (A) Vale da Couda collection sites. (B) Distribution of *Candidula* sampling sites in mainland Portugal.

The land snails of the genus *Candidula* present in Europe, from eastern Canary Islands to the Balkans and northwards to Scotland and southernmost Sweden are represented by 24 putative species. Portugal has 8 endemic species (*C. coudensis*, *C. setubalensis, C. scabiosula, C. arrabidensis, C. belemensis, C. carrapateirensis, C. codia* and *C. strucki*) from a total of 12 (*C. gigaxii, C. intersecta, C. ponsulensis, C. olisippensis*) (Holyoak & Holyoak, 2014, see Fig. 1A). Most species are hard to distinguish using conchological characters only and it takes a combination of morphological characters, such as the size of the penial flagellum or shell shape, to classify the specimens (Holyoak & Holyoak, 2014). Nevertheless, a clear, comprehensive, taxonomic assessment based on both morphological and molecular data has not been previously done. As most of the landsnails around the globe, species from the *Candidula* genus are hermaphroditic (Holyoak & Holyoak, 2014, see Fig. 1A). Most *Candidula* species prefer open and dry habitats, usually with calcareous substrate. In Portugal, species can be found in a variety of habitats, ranging from rocky limestone grasslands to sand dunes. There are records of coexisting *Candidula* species in Portugal: *C. coudensis* and *C. olisippensis* in Vale da Couda, and *C. setubalensis* and *C. arrabidensis* in Serra da Arrábida, *C. belemensis* and *C. olisippensis* in various locations of Beira Litoral, such as Serra do Sicó, and *C. gigaxii* and *C. ponsulensis* in eastern Baixo Alentejo (Holyoak & Holyoak, 2014, see Fig. 1A).

*Candidula coudensis* (Holyoak & Holyoak, 2010) is an endemic species described recently with a highly restricted geographic distribution in Vale da Couda, Leiria, Portugal. A broad-scale sampling of this region (ca. 100 km^2^) revealed that *C. coudensis* could only be found within a small area of ca. 13.5 km^2^ (Moreira, Calado & Dias, 2015).

The species can be found in open rocky limestone substrata, olive tree grounds, areas of natural vegetation, in roadside areas or even in stone-walls in nearby houses (Moreira, Calado & Dias, 2015). The extremely constrained geographic distribution is somewhat rare and there are several possible non-exclusive reasons that would justify such circumscribed distribution: (i) active dispersal may be very small with individuals hardly moving; (ii) very strict environmental and ecological requirements; (iii) present-day individuals are remnants of an older widespread haplogroup that range-contracted due to reduction of humidity levels after the Last Glacial Maximum (LGM, circa 20 k years), and/ or (iv) present-day habitat disturbance processes. We tested the following hypotheses based on premises that are likely to shape the phylogeographic structure of the land snails from Vale da Couda, putatively attributed to *C. coudensis*: (1) Vale da Couda individuals may form a monophyletic clade, indicative of a single population on a restricted area in the absence of major phylogeographic breaks (e.g., rivers or large mountains) and (2) Vale da Couda individuals are expected to show reduced levels of haplotype and nucleotide diversities, consistent with an isolated population on a limited geographical area.

Using a combination of DNA sequences (fragment of the cytochrome oxidase subunit I—COI, mitochondrial gene and of the first nuclear intron—ITS1) and geo-referenced field records of the species we sought to address the above hypotheses by revealing the genetic diversity and geographic structure of contemporary Vale da Couda individuals, and reconstructing its demographic history. Using Iberian environmental data relative to past and current conditions retrieved from public repositories, we inferred locations of the putative refugia during the LGM and provided estimates of relative environmental suitability of Vale da Couda individuals that can assist future fieldwork.

## Material and Methods

### Taxon sampling

Suitable habitat of Vale da Couda individuals consists mainly of boulders and stonewalls, and those were the preferred sites for sampling. Density was very variable, between 0.1 and one individuals per square meter on suitable habitat. The total sampling area at each of the four sites comprised a few square meters, and up to six individuals were collected within a few meters of each other. Samples for genetic assessment were lumped into four sites, which were GPS referenced.

Sampling in Vale da Couda resulted in 73 individuals collected from four different sites (Fig. 1). We received field permit from the Nature and Forests Conservation Institute (ICNF), Portugal (identifier: 81S0/201S/DCNF-LVT/DPAP) for sampling in Arrábida Natural Parque. Immediately after collection, whole shells containing the individual were stored in ethanol 70%.

### Laboratory procedures and sequence alignments

DNA was extracted from the samples using a CTAB protocol (Doyle & Doyle, 1987). Universal primers (Folmer et al., 1994) were used in PCRs to amplify 600 bp of the COI gene. PCR amplifications were performed in 25 µl total volume, using 5 µl 5X PCR Colorless Buffer (pH 8.5), 2 mM (of a 1.5 µl 25 mM MgCl2 solution), 0.2 mM (0.5 µl of a 20 mM dNTP stock), 0.2 µl 5 u/µl 1U GoTaq DNA polymerase Promega (Madison, WI, USA) and 0.2 µM (0.5 µl of a 10 µM stock) of each primer. The COI PCR profile consisted of 2 min at 95 °C, 35 cycles of 30 s at 94 °C, 30 s at 53 °C followed by an extension for 1 min at 72 °C and a final one with 5 min. ITS1 gene was amplified by PCR with forward primer ITS1—5′-TCCGTAGGTGAACCTGCGGAAGGAT-3′ (White et al., 1990) and reverse primer 5.8c—5′-TGCGTTCAAGATATCGATGTTCAA-3′ modified from Hillis & Dixon (1991). PCR amplifications were performed in 25 µl total volume, using 5 µl 5X PCR Colorless Buffer (pH 8.5), 2 mM (of a 1.5 µl 25 mM MgCl2 solution), 0.2 mM (0.5 µl of a 20 mM dNTP stock), 0.2 µl 5 u/µl 1U GoTaq DNA polymerase Promega (Madison, USA) and 0.2 µM (0.5 µl of a 10 µM stock) of each primer. The ITS1 PCR profile consisted of 3 min at 97 °C, 35 cycles of 1 min at 95 °C, 1 min at 55 °C and 2 min at 72°, followed by a final extension of 5 min at 72 °C. The PCR results were purified by ethanol precipitation (Sambrook & Russell, 2001). Sequencing was performed on an ABI 3130xl (Applied Biosystems) automated sequencer at CCMAR facilities.

COI sequences were aligned using MUSCLE (Edgar, 2004), implemented in Geneious version 7.0.4 (Kearse et al., 2012), and contained no gaps. Heterozygous ITS1 sequences were fed into Mixed Sequence Reader (MSR) (http://msr.cs.nthu.edu.tw), which separates the information from the chromatogram into a major and minor sequence, corresponding to each allele, while comparing the sequence information with a given reference sequence (Chang et al., 2012). Major and minor sequences for each sample were recovered and posteriorly aligned using MAFFT default options (Katoh & Standley, 2013).

### Population genetics

Molecular diversity indices, including nucleotide (*π*) (Nei, 1987) and haplotype (*h*) (Nei & Tajima, 1981) diversities, were estimated using DnaSP v5.10 (Librado & Rozas, 2009). To evaluate the level of population differentiation among four Vale da Couda sites, we used *F*_*ST*_ genetic fixation (Weir & Cockerham, 1984) and *D*_*est*_ genetic differentiation (Jost, 2008) statistics estimated with the modelling package 1.9.5 (Keenan et al., 2013). The variance of each statistic was assessed through the calculation of 10 000 pairwise bootstrapped 95% confidence limits using a bias corrected method that basically re-centers the confidence interval (CI) around the initial parameter estimate. We employed both genetic estimators as they present advantages and drawbacks in quantifying population structure (for a discussion see Bird et al., 2011; Jost, 2008; Meirmans & Hedrick, 2011; Ryman & Leimar, 2009; Whitlock, 2011).

Phylogeographic relationships among haplotypes of COI and ITS1 alleles were represented using the Median Joining Network method (Bandelt, Forster & Röhl, 1999) implemented in Network (version 4.6.1.0; fluxus-engineering.com) that infers the most parsimonious branch connections between sequences. Net divergences between and within mtDNA and nuclear DNA haplogroups were calculated using MEGA6 (Tamura et al., 2013) using the Tamura-Nei model (Tamura & Nei, 1993) for both data sets.

### Taxonomic context

To place the Vale da Couda samples in a broader phylogenetic context and to ascertain the non-monophyly of the individuals from Vale da Couda (given the very distant haplogroups found—see Results section below), putative *Candidula spp.* individuals were collected in different locations (Table S1) to ascertain the taxonomic status of the individuals from Vale da Couda. It was not, however, our intention to produce a complete and thorough phylogeny for the genus *Candidula*. We followed Holyoak & Holyoak (2014) taxonomy to identify some specimens based on morphology. The partial sequences of the mitochondrial (mtDNA) COI gene including 73 *Candidula* from Vale da Couda, produced a data set of 464 nucleotide positions. The Akaike Information Criterion (Akaike, 1974) implemented in Modeltest selected the K81uf + I + G as the evolutionary model that best fits the data set. Since this model is not available in PhyML v.3.0 (Guindon & Gascuel, 2003), we selected the second best-fit model, the HKY + G. The selected model and model parameters were used in the Maximum Likelihood (ML) analysis performed with PhyML v.3.0 (Guindon & Gascuel, 2003). The robustness of the inferred trees was tested by nonparametric bootstrapping (BP) using 1000 pseudoreplicates. ML analysis was carried out at the Mobyle platform (http://mobyle.pasteur.fr/cgi-bin/portal.py).

### Environmental niche modelling

The study area was the Iberian Peninsula. Bioclimatic variables for current conditions were retrieved from WorldClim dataset (Hijmans et al., 2005) in 30 arc seconds (∼1 km), resolution used for all modelling analyses (Table S2). In addition, because of the species preference for limestone soils, where it is most frequently found (Moreira, Calado & Dias, 2015), we extracted the distribution of carbonate sedimentary rocks (e.g., limestone, dolomite and marl) from a global lithological map (Hartmann & Moosdorf, 2012). The percentage of this lithological class was calculated for each grid cell of the Iberian Peninsula to be included as a quantitative variable in the models. Assuming that no significant change on the Iberian distribution of continental rock lithology was produced during the last 21k years, we used the same lithological variable for the LGM projections. LGM climatic variables were obtained from Schmatz et al. (2015) in 30 arc seconds resolution according to four general circulation models (GCMs) pertaining to the Coupled Model Intercomparison Project (CMIP5: http://cmip-pcmdi.llnl.gov/cmip5/): CCSM, CNRM, IPSL and MIROC3.2.

The model was built based on 89 presence records, identified by Moreira, Calado & Dias (2015), which fall in 33 different 1 km^2^ cells. As the distribution of this recently discovered species is restricted (Moreira, Calado & Dias, 2015), the spatial autocorrelation of the variables is high, thus we limited the number of variables to a maximum of three to avoid over-parameterization. To select the variables, we firstly performed a Pearson correlation analysis using a threshold of *r* = —±0.7—. Then, we performed an Ecological Niche Factor Analysis (ENFA, Hirzel et al., 2002) with the preselected uncorrelated variables. ENFA computes factors accounting for the position of the occurrence data in the multidimensional environmental space of the study area. These factors describe the environmental niche of the species by computing the distance between the mean habitat for the species in relation to the study area (marginality) and the variance of the species’ niche (specialization). Thus, ENFA can be an exploratory analysis to select the most relevant variables describing the niche of the species (see e.g., Chefaoui et al., 2015; Lobo, Jiménez-Valverde & Hortal, 2010).

To model the distribution of Vale da Couda individuals under current and LGM conditions we used Maxent (Phillips, Anderson & Schapire, 2006), a maximum entropy algorithm which uses presence and background data. This technique allows a “clamping” process, which handles predictors outside the training range as if they were at the limit. We selected ten times more background points than presences at random in order to set a prevalence of 0.1, as this proportion was used before with good results (e.g., Chefaoui et al., 2015; Chefaoui & Lobo, 2008). We split data into a training (80%) and a test set (20%) to perform a cross-validation during 100 iterations. To validate the models, we obtained the area under the receiver operating characteristic (ROC) curve (AUC), the sensitivity (presences correctly predicted) and the specificity (absences correctly predicted) scores using three different thresholds for validation: the prevalence (=0.1), the value which maximizes the sum of the sensitivity and specificity, and the highest threshold at which there is no omission. An ensemble of predictions was obtained for current conditions by computing the average of the 100 iterations. For LGM projection, we produced a hindcast using the average of the four GCMs. All analyses were performed in R (R Development Core Team, 2016) using “adehabitat” and “dismo” packages.

## Results

### Population genetics

**Figure 2:**
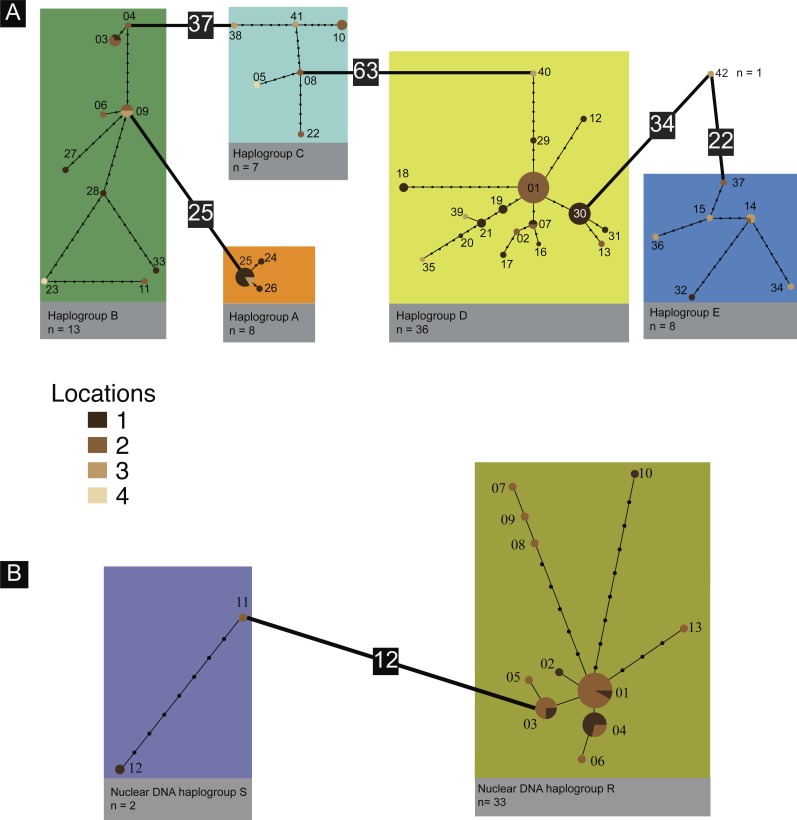
Haplotype networks. (A) MtDNA COI statistical parsimony haplotype network for Vale da Couda individuals. (B) Nuclear ITS1 statistical parsimony haplotype network for Vale da Couda individuals. Each branch represents one inferred mutational step; small black circles on branches represent additional inferred mutational steps; numbers in black squares denote more than twenty mutations.

**Figure 3:**
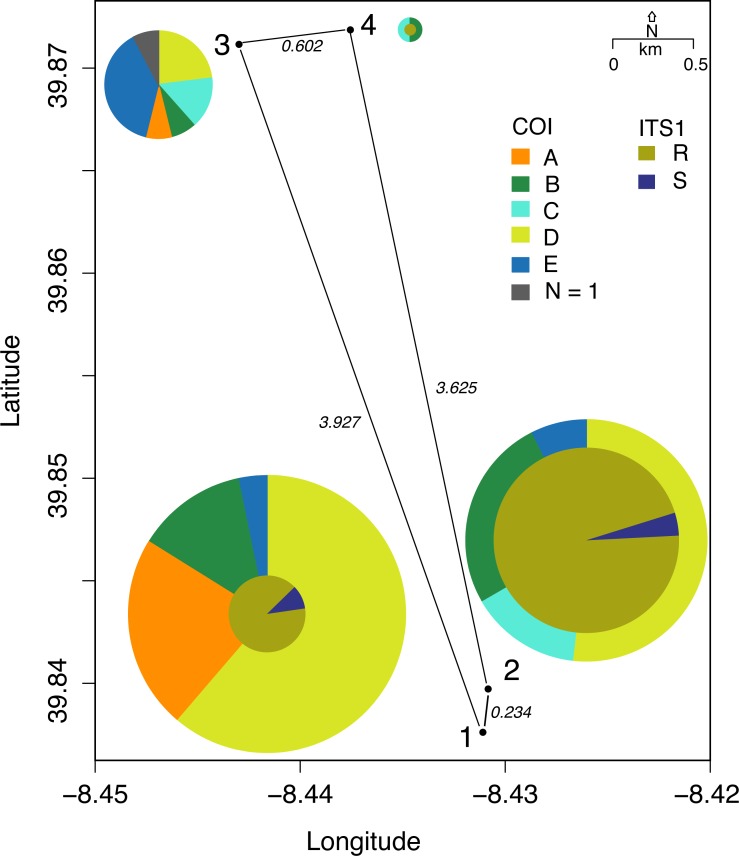
Distribution of mtDNA lineages in Vale da Couda sites. Numbers represent sites; numbers in italic represent distance in km between sites. Size of circles is proportional to the number of individuals. Colors depicting haplogroups are the same as in Fig. 2.

MtDNA sequence data of 73 putative *C. coudensis* individuals from Vale da Couda generated a 560-bp fragment alignment with a total of 142 polymorphic sites, 124 of which were parsimony informative. These polymorphisms defined 42 haplotypes with an overall haplotype diversity and mean nucleotide diversity of *h* = 0.964 ± 0.011 and *π* = 0.084 ± 0.004, respectively (Table 1A). These haplotypes were organized into five main divergent haplogroups, with 22 to 63 mutation steps apart (Fig. 2A). Net sequence divergence between haplogroups ranged from 11.8 to 47.5%, while within net sequence divergence ranged from 0.1–2.1% (Fig. S1). A large proportion of individuals (45%) possess unique haplotypes. The majority of haplotypes (88%) is found in only one location (i.e., ‘private’ haplotypes), and only five haplotypes are shared among sites (12%). Despite the existence of these distinct haplogroups, there is no obvious phylogeographic pattern and no evidence for closely related haplotypes (i.e., same haplogroup) to come from the same location (Figs. 2 and 3).

**Table 1:** Vale da Couda lineages and sites statistics. Vale da Couda lineages, sample sizes and summary statistics for COI and ITS1 sequence fragments (A). Vale da Couda site sample sizes, lineages present and summary statistics for COI and ITS1 (B).

(A)										
Lineages	COI					ITS1				
	*N*	*Nh*	Locations	*h* ± s.d.	*π* ± s.d.	*N*	*Nh*	Locations	*h* ± s.d.	*π* ± s.d.
A	8	3	1, 3	0.464 ± 0.040	0.001 ± 0.000					
B	13	9	1, 2, 3, 4	0.936 ± 0.051	0.018 ± 0.002					
C	7	6	2, 3, 4	0.952 ± 0.096	0.010 ± 0.096	2	2	1,2	1.000 ± 0.500	0.016 ± 0.008
D	36	17	1, 2, 3	0.889 ± 0.001	0.009 ± 0.001	33	11	1,2	0.799 ± 0.054	0.006 ± 0.001
E	8	6	1, 2, 3	0.893 ± 0.111	0.013 ± 0.003					

(B)										
Locations	COI					ITS1				
	*N*	*Nh*	Lineages	*h* ± s.d.	*π* ± s.d.	*N*	*Nh*	Lineages	*h* ± s.d.	*π* ± s.d.
1	31	18	A, B, D, –	0.927 ± 0.031	0.075 ± 0.008	10	6	R, S	0.778 ± 0.137	0.011 ± 0.005
2	27	15	B, C, D, –	0.863 ± 0.062	0.081 ± 0.007	25	10	R, S	0.763 ± 0.007	0.007 ± 0.002
3	13	12	A, B,C, D, –	0.987 ± 0.035	0.091 ± 0.009					
4	2	2	B, C	1.000 ± 0.500	0.083 ± 0.042					

**Notes.**

TITLE *N* Sample size *Nh* number of haplotypes *h* haplotype diversity *π* nucleotide diversity s.d. standard deviation

PCR amplification of the nuclear intron was only successful in 35 individuals from Vale da Couda, generating a 503-bp fragment alignment with a total of 39 polymorphic sites, 17 of which were parsimony informative. The sequences defined 13 haplotypes with an overall haplotype diversity and mean nucleotide diversity of *h* = 0.822 ± 0.050 and *π* = 0.009 ± 0.002 respectively (Table 1A). These haplotypes constitute two haplogroups separated by 12 mutation steps (Fig. 2B). Net sequence divergence between haplogroups was 2.3%, while within net sequence divergence ranged from 0.6–1.6%. Only 26% of the individuals have a unique haplotype. Of the total 13 haplotypes, 10 were private and three (23%) were shared between locations.

MtDNA haplotypes were unevenly distributed among the four sampling sites (Fig. 3). Sites 1 and 3 have representatives from all groups while site 2 has no representation of haplogroup C. In site 4 only haplogroups B and D are represented. The two ITS-1 haplogroups are just present in two sampled sites, 1 and 2 (Fig. 3). We found no association between nuclear DNA and mtDNA haplogroups (Table S3). ITS-1 sequences were organized into two haplogroups (R and S), with disproportional representation among them (Fig. 3).

### Phylogenetic estimation

**Figure 4:**
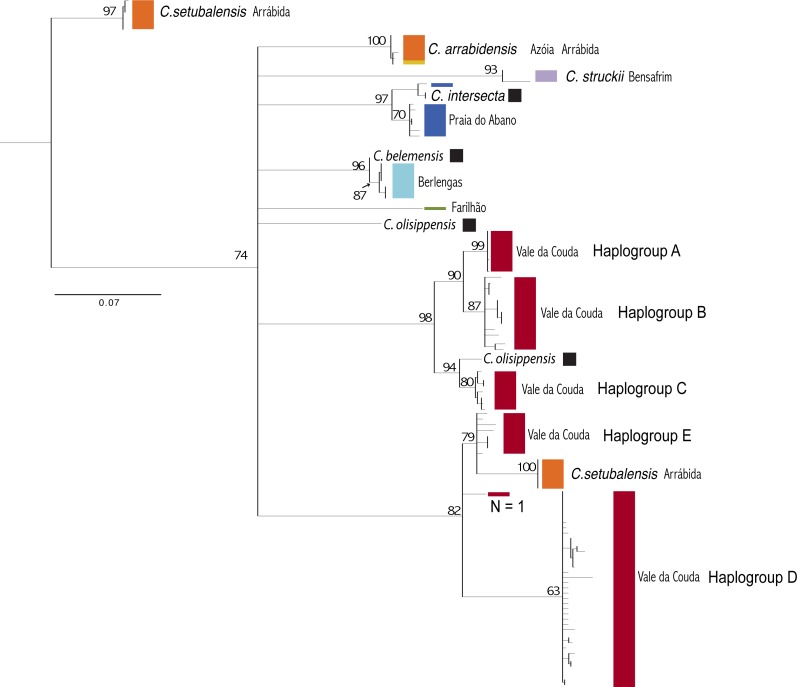
Phylogenetic relationships between *Candidula* individuals from Vale da Couda (in red) and other locations in Portugal. Black squares represent individuals identified morphologically and anatomically. Colours of locations as in Fig. 1. Outgroups removed from figure for illustrative purposes.

Results from the haplotype network suggest that the Vale da Couda individuals are not monophyletic, given the extreme genetic distance between haplogroups. The ML analysis (−ln *L* =  − 1622.11) based on the COI data set yielded the topology depicted in Fig. 4. Specimens from Vale da Couda grouped into two main clades that did not cluster together. One clade included three haplogroups supported by high BP values (A, B, C). Haplogroup C grouped with specimens assigned to *C. olisippensis*. The other clade included haplogroup D and Haplogroup E from Vale da Couda that showed an unresolved phylogenetic position. These specimens grouped with *C. setubalensis* from Arrábida (Fig. 4).

### Niche modelling

**Figure 5:**
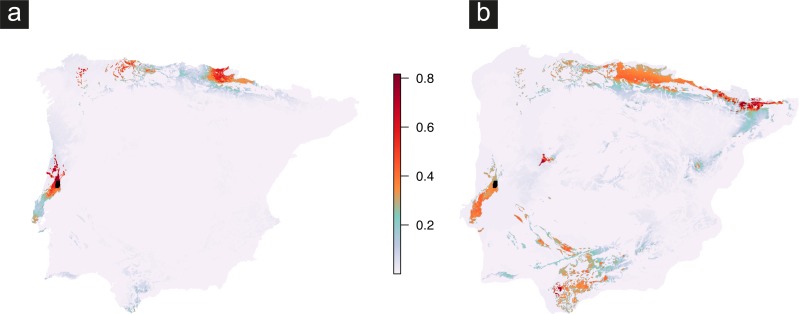
Predicted geographic distribution built with presences of Vale da Couda individuals and based on (A) current climate, and (B) Last Glacial Maximum (LGM) conditions. Colour scale represents high probability of occurrence in red and low levels in blue. Black dots represent the present-day known occurrences in the Vale da Couda. Ice sheet existing during LGM in the north of the Iberian Peninsula is not depicted.

Eight uncorrelated climatic variables were used to perform ENFA analysis, which finally distinguished lithology, isothermality (BIO3), and the annual precipitation (BIO12) as the three most relevant variables defining the niche for Vale da Couda lineages (Table 2). ENFA marginality factor revealed that the lithology (grid cells with high percentage of carbonate sedimentary rocks) was the most relevant predictor of its distribution, an expectable result as the species has been found exclusively on limestone (Moreira, Calado & Dias, 2015). Besides, ENFA showed that the species has a preference for locations where the isothermality and the annual precipitation are higher than the mean conditions of the Iberian Peninsula (Table 2). Maxent models produced a strong discrimination between presence and background data regardless of the threshold used (Table 3). Overall validation scores of models calibrated under current conditions were: mean AUC =0.981 ± 0.002, mean sensitivity =0.979 ± 0.017, and mean specificity =0.982 ± 0.012 (Table 3). The resulting ensemble for the current distribution showed two main areas with high probability of presence of Vale da Couda lineages: (1) one around the presently known distribution, and (2) different patches at the north of the Iberian Peninsula (Fig. 5A). LGM projection indicates that past distribution of suitable habitats could have been wider, with also appropriate conditions in the Andalusian region and in a smaller area in the Central System (Fig. 5B).

**Table 2:** Environmental Niche Factor Analysis (ENFA) results showing marginality and specialization factors scores. The three variables with higher marginality scores (in bold) were selected for subsequent analyses.

Variable	Marginality	Specialization
bio1	0.19	0.40
bio3	**0.47**	0.00
bio7	−0.40	0.10
bio8	−0.10	0.05
bio12	**0.45**	0.07
bio9	0.07	−0.91
bio17	−0.10	0.03
lithology	**0.59**	0.00

**Table 3:** Summary of Maxent models. Mean AUC, sensitivity and specificity scores obtained from the 100 Maxent models according to the three thresholds used. (Spec_sens: threshold that maximizes the sum of the sensitivity and specificity).

Model validation	Threshold	Mean ± standard deviation
AUC	Prevalence	0.983 ± 0.007
	No omission	0.980 ± 0.019
	Spec_sens	0.980 ± 0.018
Sensitivity	Prevalence	0.999 ± 0.006
	No omission	0.971 ± 0.037
	Spec_sens	0.969 ± 0.038
Specificity	Prevalence	0.968 ± 0.013
	No omission	0.989 ± 0.020
	Spec_sens	0.990 ± 0.010

## Discussion

Mitochondrial sequence data used in this study produced a Vale da Couda complex phylogeny, with highly divergent clades that reject the monophyly of *C. coudensis*. Results presented are surprising because they reflect higher genetic variability than expected in species with restricted geographic distributions, low dispersal potential and large estimated population sizes. Despite our results add to a growing list of taxa showing limited distribution and high genetic diversity (e.g., Coates, Tischler & McComb, 2006; Ellis, Pashley & McCauley, 2006; Gevaert et al., 2013; Young, Boyle & Brown, 1996), sound explanations for the phenomenon are not trivial.

### Population genetics

Studies have shown populations with high genetic structure existing during the LGM in the Iberian Peninsula (Gómez & Lunt, 2007). The particular geographical characteristics of this region (e.g., the existence of multiple mountain ranges with an east–west orientation creating a wide array of microclimatic changes or the influence of both the North Atlantic and the Mediterranean Sea) foster the perfect conditions for the isolation of populations creating the “refugia within refugia” (Gómez & Lunt, 2007). Even though our LGM distribution model suggests a larger distribution area for Vale da Couda lineages, it is possible that *Candidula* populations have endured geographical fragmentation at a micro-geographical level. Due to effects of genetic drift in geographically limited species we would expect that our results showed lineages from Vale da Couda to be genetically depauperated but each sampled location displayed high levels of genetic diversity (Table 1B). The five highly-divergent mtDNA clades found in Vale da Couda may have resulted from multiple colonization events by different individuals of the same species that extended their distribution towards more southern locations during the LGM.

The maintenance of diversity in rare species can be explained by the existence of a large effective population size (Ellstrand & Elam, 1993). The 100,000 to 300,000 *Candidula* individuals estimated to exist in Vale da Couda (Moreira, Calado & Dias, 2015) may represent a large population size considering its putative circumscribed distribution (c.a. 13.5 km^2^). Nevertheless, the following assumptions generated from the large population size premise must be considered:

(1) Retention of ancestral polymorphisms. Incomplete lineage sorting is higher in large populations, increasing the probability of sampling more intermediate haplotypes. Regardless the large population size of *Candidula* from Vale da Couda, our network (Fig. 2) shows an absence of intermediate haplotypes and widely separated haplogroups;
(2) High mutation rates. Possible explanation for the observed diversity could be the occurrence of high mutation rates, as reported in other groups of land snails (Chiba, 1999; Davison, Blackie & Scothern, 2009; Haase et al., 2003; Thomaz, Guiller & Clarke, 1996). High mutation rates would generate a large number of different haplotypes reducing the probability of sampling the same haplotype at more than one site. This premise is largely supported by our results given that only five (12%) of the 42 haplotypes sampled in the present work were found in more than one location;
(3) Common ancestry in a restricted area. Because *Candidula* living individuals in Vale da Couda are present in a highly restricted geographic area (13.5 km^2^), we would expect that most of the individuals would share a common ancestry and generate groups of rather closely related haplotypes given their putative low dispersal abilities. However, our haplotype network showed a large number of gaps within each haplogroup, not supporting the shared ancestry hypothesis;
(4) Existence of cryptic species. Finally, if each haplogroup represents a cryptic species, we would expect smaller population sizes with more related individuals within each group and fewer gaps within haplogroups. This expectation is not met by our results considering the large number of gaps separating haplotypes of the same haplogroup (Fig. 2).

### Environmental niche modelling

Two distribution models were produced for specimens found in Vale da Couda: a present-day model and a LGM model (*circa* 20 k years). The LGM model shows a wider area that extends to the south with higher probability of occurrence compared with the present-day distribution of the individuals from Vale da Couda (Fig. 5B). This predicted distribution implies a co-occurrence between Vale da Couda lineages and other species of the genus (e.g., *C. setubalensis* and *C. olissipensis*) currently occupying these southern locations. The differences between the paleo-model and the contemporary model are somewhat unexpected considering that most of the northern hemisphere terrestrial organisms have contracted their geographic distributions to the south during harsher glacial climate conditions, and have expanded their distribution by re-colonizing former northern territories after deglaciation (Hewitt, 1999). Mountainous regions of the north of the Iberian Peninsula (i.e., Pyrenees and Cantabrian Range) are known to have been covered by ice during Pleistocene glaciations, though the precise position of the ice sheet in the LGM remains uncertain (see e.g., Palacios et al., 2015). Thus, most of those northern regions found suitable by our LGM model could not have been occupied by these terrestrial land snails because of the existent ice sheet before deglaciation.

According to ENFA results (Table 2) the present-day distribution of lineages from Vale da Couda is mainly driven by the presence of carbonate-dominated lithological units under rainy and isothermal climatic conditions. These specific requirements seem to be in agreement with those shown by other terrestrial mollusc species (Hermida, Ondina & Rodriguez, 2000; Kadmon & Heller, 1998; Tattersfield et al., 2001; Tsoar et al., 2007).

Given the putative low dispersal capacity of this group, the most plausible hypothesis is that during Quaternary glaciations Vale da Couda lineages might have dispersed towards more suitable habitat located in south-central Portugal (Lisbon and northeast of Lisbon, including Leiria), as suggested by LGM hindcast. A postglacial change of climatic conditions towards lower precipitation in the Lisbon area may have caused Vale da Couda lineages contraction to its actual distribution using the suitable Mesozoic calcareous rock as a corridor. Although we have addressed some common hindcasting uncertainties by using different GCMs and a clamping mask hindcast approach, we could not resolve the lack of accurate lithological data for emerged coastal land in the LGM. More appropriate habitats not depicted in our models could have existed in regions near the coast.

### Biogeographic scenario

Given the uncertainties regarding the possible explanations of the genetic results, we will not dwell on putative alternative biogeographic scenarios to explain the high genetic diversity found in Vale da Couda. We will, nevertheless, propose a hypothesis that relies on species co-existence in more southern locations followed by a northern dispersal tracking the species optimal thermal, humidity and soil physical conditions. This co-existence is plausible given the fact that *C. setubalensis* and *C. arrabidensis* occur in sympatry, as well as *C. olisippensis* and *C. coudensis* (Holyoak & Holyoak, 2014) and share habitat requirements with Vale da Couda lineages. Moreover, Roucoux et al. (2001) shows low but fluctuating tree pollen through the LGM, along with abundant grass and some herb pollen, indicating likely widespread suitability of the grassy habitats for *Candidula* species throughout the LGM. Similar events have already been detected for another land snail species (Harl et al., 2014; Sauer & Hausdorf, 2010; Shimizu & Ueshima, 2000). After the LGM, environmental conditions during deglaciation were such that promoted northward dispersal of land snails and the establishment of populations in locations of suitable isothermality and precipitation like Vale da Couda. *C. setubalensis* and *C. arrabidensis* maintained a southern distribution, in the Setubal Peninsula. Specifically, we hypothesize that Pleistocene conditions may have isolated populations into pockets of suitable habitats in more southern locations, which promoted population differentiation and intra-specific diversification without apparent geological barriers.

### Conclusions

The genetic survey presented here revealed the existence of four main mitochondrial lineages in Vale da Couda (previously attributed to a single species) with independent evolutionary histories and exhibiting extremely narrow geographic ranges. These results do not corroborate previous morphological studies that considered the existence of a single species, *Candidula coudensis* in the studied area. The high genetic diversity and the haplotype network inherent characteristics (haplogroups and haplotypes within haplogroups separated by a relatively large number of mutations) cannot be fully explained with present data. LGM hindcasts revealed the existence of putative glacial refugia south of the current distribution of the lineages of Vale da Couda. These findings have implications for the understanding of the genetic characteristics of rare and endemic species. From a conservation perspective, Vale da Couda lineages do not seem to be endangered, with high genetic diversity within and between lineages maintained by putative large effective population sizes.

##  Supplemental Information

10.7717/peerj.3069/supp-1 Table S1 Sample location and statistics Sample location and summary statistics for the genus *Candidula*.****

10.7717/peerj.3069/supp-2 Table S2 Bioclimatic variables Bioclimatic variables for current conditions retrieved from WorldClim dataset (Hijmans et al., 2005).

10.7717/peerj.3069/supp-3 Table S3 List of individuals, COI and ITS1 haplotypes, haplogroups and location sites

10.7717/peerj.3069/supp-4 Figure S1 Evolutionary divergence between haplogroups Estimates of net evolutionary divergence between haplogroups (axis on the left, dark grey bars ± standard deviation) and within lineages (axis on the right, light grey bars ±standard deviation), based on Tamura-Nei distances.

## Additional Information and Declarations

### Field Study Permissions

The following information was supplied relating to field study approvals (i.e., approving body and any reference numbers): We received a field permit from the Nature and Forests Conservation Institute (ICNF), Portugal (identifier: 81S0/201S/DCNF-LVT/DPAP) for sampling in Arrábida Natural Parque.

### DNA Deposition

The following information was supplied regarding the deposition of DNA sequences: GenBank: KX766219–KX766326, KX766327–KX766332, KX766333–KX766367.

### Data Availability

The following information was supplied regarding data availability: The research in this article did not generate, collect or analyse any raw data or code.
