# Supplementary material for: High unexpected genetic diversity of a narrow endemic terrestrial mollusc
Source: PeerJ. 2017 Mar 16;5:e3069. doi: 10.7717/peerj.3069 (PMC5357342; doi:10.7717/peerj.3069)
Supplement: Table S1 — Sample location and summary statistics for the genus Candidula. [file peerj-05-3069-s001.docx]

| **Location** | **Code** | **Long** | **Lat** |  | ***N*** | ***nh*** | ***h ± s.d*** | | | π **± s.d.** | | |
| --- | --- | --- | --- | --- | --- | --- | --- | --- | --- | --- | --- | --- |
| Arrábida | AR | 38.5 | -9.0 |  | 17 | 9 | 0.860 | ± | 0.068 | 0.108 | ± | 0.009 |
| Azoia, Espichel | AZ | 38.4 | -9.2 |  | 3 | 3 | 1.000 | ± | 0.272 | 0.105 | ± | 0.047 |
| Berlengas | BE | 37.2 | -8.7 |  | 7 | 2 | 0.571 | ± | 0.119 | 0.004 | ± | 0.000 |
| Bensafrim | BS | 39.4 | -9.4 |  | 4 | 3 | 0.833 | ± | 0.222 | 0.116 | ± | 0.054 |
| Farilhão | FA | 39.5 | -9.5 |  | 1 | 1 | 0.000 |  |  | - |  | - |
| Praia do Abano | AB | 38.7 | -9.5 |  | 10 | 9 | 0.978 | ± | 0.054 | 0.025 | ± | 0.006 |
| Vale da Couda | VC | 39.8 | -8.4 |  | 73 | 42 | 0.964 | ± | 0.011 | 0.084 | ± | 0.004 |
| **Total** |  |  |  |  | 115 | 69 | 0.972 | ± | 0.009 | 0.122 | ± | 0.004 |

*N*: number of individuals; *Nh*: number of haplotypes; *h*: haplotype diversity; ?: nucleotide diversity; *s.d.*: standard deviation.
