# Supplementary material for: High unexpected genetic diversity of a narrow endemic terrestrial mollusc
Source: PeerJ. 2017 Mar 16;5:e3069. doi: 10.7717/peerj.3069 (PMC5357342; doi:10.7717/peerj.3069)
Supplement: Table S2 — Bioclimatic variables for current conditions retrieved from WorldClim dataset (Hijmans et al., 2005). [file peerj-05-3069-s002.docx]

Supplemental information

**Table S1.**  Bioclimatic variables for current conditions retrieved from WorldClim dataset (Hijmans et al 2005).

| **Code** | **Bioclimatic variables** | **Initial climatic factor** |
| --- | --- | --- |
| Bio1 | Annual Mean Temperature | T |
| Bio2 | Mean Diurnal Range [Mean of monthly (max temp - min temp)] | T |
| Bio3 | Isothermality [(Bio2/Bio7)*100] | T |
| Bio4 | Temperature Seasonality [standard deviation *100] | T |
| Bio5 | Max Temperature of Warmest Month | T |
| Bio6 | Min Temperature of Coldest Month | T |
| Bio7 | Temperature Annual Range [Bio5-Bio6] | T |
| Bio8 | Mean Temperature of Wettest Quarter | T + R |
| Bio9 | Mean Temperature of Driest Quarter | T + R |
| Bio10 | Mean Temperature of Warmest Quarter | T |
| Bio11 | Mean Temperature of Coldest Quarter | T |
| Bio12 | Annual Precipitation | R |
| Bio13 | Precipitation of Wettest Month | R |
| Bio14 | Precipitation of Driest Month | R |
| Bio15 | Precipitation Seasonality [Coefficient of Variation] | R |
| Bio16 | Precipitation of Wettest Quarter | R |
| Bio17 | Precipitation of Driest Quarter | R |
| Bio18 | Precipitation of Warmest Quarter | T + R |
| Bio19 | Precipitation of Coldest Quarter | T + R |

T, Temperature ; R, Rainfall ; T + R, Both
