# Supplementary material for: High unexpected genetic diversity of a narrow endemic terrestrial mollusc
Source: PeerJ. 2017 Mar 16;5:e3069. doi: 10.7717/peerj.3069 (PMC5357342; doi:10.7717/peerj.3069)

**Figure S1.** Estimates of net evolutionary divergence between haplogroups (axis on the left, dark grey bars ± standard deviation) and within lineages (axis on the right, light grey bars ± standard deviation), based on Tamura-Nei distances.

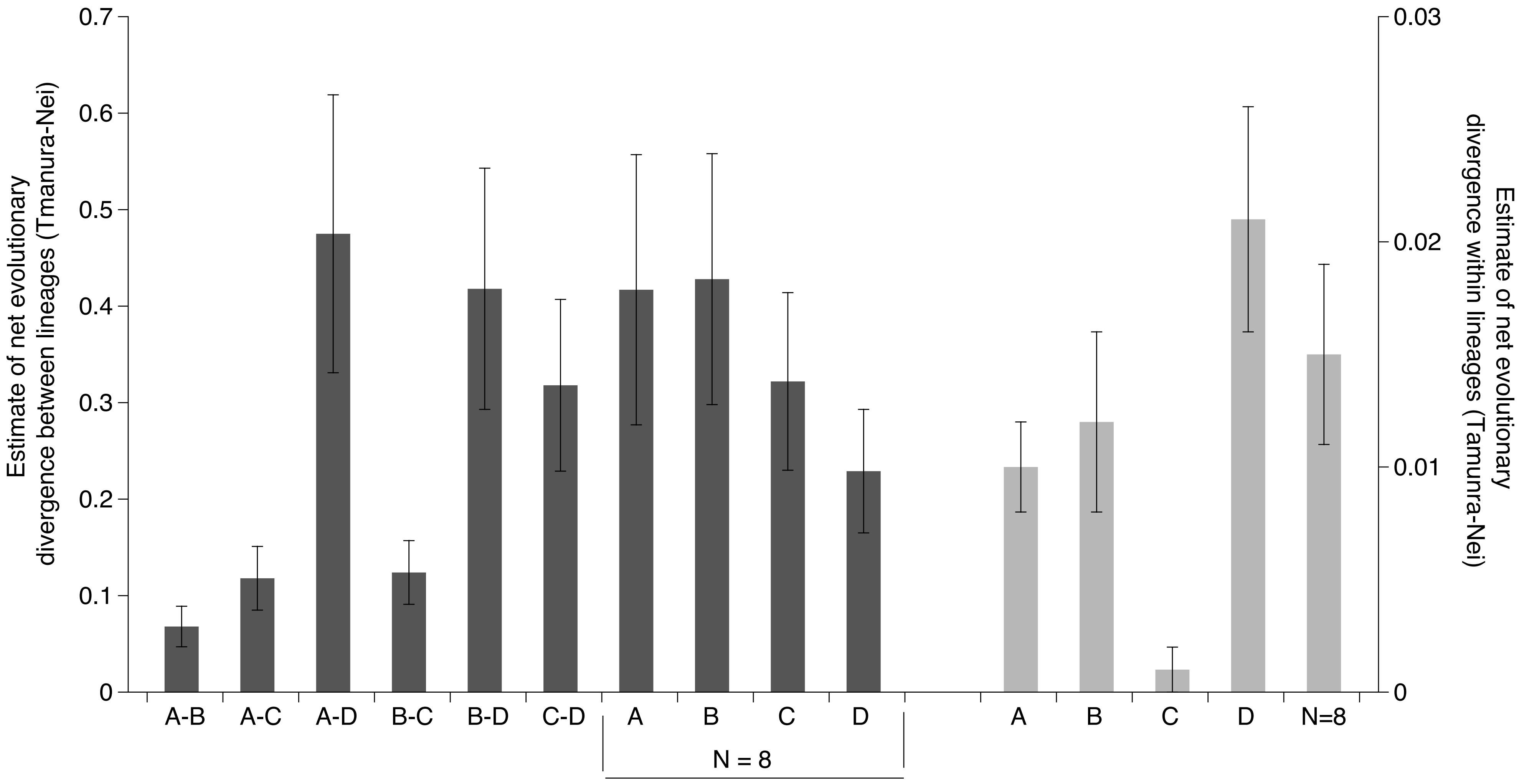

Supplement: Figure S1 — Estimates of net evolutionary divergence between haplogroups (axis on the left, dark grey bars ± standard deviation) and within lineages (axis on the right, light grey bars ±standard deviation), based on Tamura-Nei distances. [file peerj-05-3069-s004.pdf]
